# The oxidative balance and stopover departure decisions in a medium- and a long-distance migrant

**DOI:** 10.1186/s40462-023-00372-7

**Published:** 2023-02-06

**Authors:** Cas Eikenaar, Alessia Ostolani, Vera Brust, Thiemo Karwinkel, Heiko Schmaljohann, Caroline Isaksson

**Affiliations:** 1 Institute of Avian Research “Vogelwarte Helgoland”, 26386 Wilhelmshaven, Germany; 2 Department of Biology, University of Pisa, 56126 Pisa, Italy; 3 Institute for Biology and Environmental Sciences (IBU), Carl von Ossietzky University of Oldenburg, 26129 Oldenburg, Germany; 4 Department of Biology, Lund University, 223 62 Lund, Sweden

**Keywords:** Departure, Migration, Physiology, Radio-telemetry, Stopover

## Abstract

**Background:**

Birds have extremely elevated metabolic rates during migratory endurance flight and consequently can become physiologically exhausted. One feature of exhaustion is oxidative damage, which occurs when the antioxidant defense system is overwhelmed by the production of damaging reactive oxygen species (ROS). Migrating birds have been shown to decrease the amount of oxidative lipid damage during stopovers, relatively stationary periods in between migratory flights. It has therefore been argued that, in addition to accumulating fuel, one of the functions of stopover is to restore the oxidative balance. If this is so, we would expect that migrating birds are unlikely to resume migration from stopover when they still have high amounts of lipid damage.

**Methods:**

To test this hypothesis, we measured parameters of the oxidative balance and related these to stopover departure decisions of song thrushes (*Turdus philomelos*) and northern wheatears (*Oenanthe oenanthe*), a medium- and long-distance songbird migrant, respectively. We measured malondialdehyde (MDA) concentration, a biomarker for oxidative lipid damage, and total non-enzymatic antioxidant capacity (AOX), an overall biomarker of protection against ROS. Stopover departure decisions were determined using a fully automated telemetry system set-up on our small island study site.

**Results:**

The decision to resume migration was not related with MDA concentration in either study species, also not when this was corrected for circulating fatty acid concentrations. Similarly, AOX did not affect this decision, also not when corrected for uric-acid concentration. The time within the night when birds departed also was not affected by MDA concentration or AOX. However, confirming earlier observations, we found that in both species, fat individuals were more likely to depart than lean individuals, and fat northern wheatears departed earlier within the night than lean conspecifics. Northern wheatears additionally departed earlier in spring with more southerly winds.

**Conclusions:**

We found no support for the idea that stopovers departure decisions are influenced by parameters of the oxidative balance. We discuss possible reasons for this unexpected finding.

## Background

In most avian migrants, the temporal schedule and associated speed of migration is strongly dependent on the number and duration of stopovers [47, 49]. Stopovers have various functions [37, 41, 50] of which efficient fuel accumulation is pivotal to successful completion of migration. Fuel is required for migratory endurance flights, and, accordingly, many studies observed that lean individuals are less likely to depart from stopover than fat individuals (e.g. [8, 11, 26, 38, 44]). Other aspects of a migrant’s physiological state and their potential effects on stopover behaviour (departure decisions) have received far less attention. This is somewhat surprising as migratory flights are performed at incredibly high metabolic rates [28] for hours to even days on end [25, 36], and thus are physiologically extremely demanding. One physiological consequence of elevated metabolic rates is an increase in reactive oxygen species (ROS). Vertebrates, including birds, have evolved a complex antioxidant defence system to avoid or minimize the damaging effects that ROS can have on lipids, proteins and DNA [2, 7, 14, 22, 24]. Yet, even though antioxidant defences are upregulated during migration [18, 29, 32], endurance flight has been shown to cause oxidative damage to lipids and proteins [13, 15, 19, 32]. With the negative effect of endurance flight on birds’ oxidative balance between ROS production and antioxidant defense, it seems plausible that migrants need to recover this balance at stopover. Indeed, recently it was shown that migrants can rapidly reduce the amount of oxidative lipid damage while at stopover [12, 20, 51], thereby clear their body from oxidative damage and probably recovering their oxidative balance. The role of antioxidants in recovery of the oxidative balance at stopover is currently unclear. Non-enzymatic antioxidant capacity either is unchanged [20, 51] or increases [12], while enzymatic antioxidant capacity has been observed to decrease during stopover [12].

Analogous to fuel accumulation, one could argue that recovery of the oxidative balance is a function of stopover; where fuel stores decrease while flying and increase during stopover, oxidative damage increases while flying and decreases during stopover. What is missing to complete the argument are studies showing that, comparable to migrants’ fuel stores, also the amount of oxidative damage affects migrants’ stopover departure decisions. A similar argument could be constructed for migrants’ antioxidant defences, however, the dynamics of antioxidant capacity during the flight-stopover cycle are not as clear as for oxidative damage; it is unknown whether antioxidant capacity changes during flight and ambiguous if or how it changes during stopover (see above).

The aim of the current study was to provide information regarding the missing piece of the argument that recovery of the oxidative balance is a function of stopover. Therefore, we linked migrants’ plasmatic oxidative lipid damage (malondialdehyde (MDA) concentration) and total non-enzymatic antioxidant capacity (AOX) to stopover departure decisions. We blood-sampled and radio-tagged both a nocturnal long-distance migrant, the northern wheatear (*Oenanthe oenanthe*, wheatears hereafter), and a nocturnal medium-distance migrant, the song thrush (*Turdus philomelos*). Departures from stopover were determined with a fully automated radio-telemetry system constructed at our small island study site. MDA is a frequently used biomarker of current and rapid oxidative damage to lipids. Therefore, in the context of short stopovers it is more informative and reliable as an indicator of ROS exposure and antioxidant efficiency than, for example, DNA damages which require long-term exposure to ROS before accumulation can be detected. Also, MDA is a secondary product of peroxidation of polyunsaturated fatty acids [23], and migrating birds use fatty acids to fuel their flights [27, 31]. AOX is a reliable and general (non-specific) marker of antioxidant capacity of plasma. AOX thus provides a good overall marker of protection against ROS and of recent ROS exposure compared to specific assays of individual antioxidants. We expected that in both species, stopover departure probability decreases with MDA concentration. Additionally, we expected the birds to depart earlier within the night (and thus presumably to make longer flights, cf. Müller et al. [42]) when having lower MDA concentration. Formulating expectations for AOX is more challenging. On the one hand, migrants have been hypothesized to build ‘prophylactic’ antioxidant capacity before a long-distance flight [51]. If so, with high AOX, we would expect increased departure likelihood and earlier nocturnal departure time. On the other hand, high AOX may be a response to high ROS exposure [14, 22]. If so, with high AOX, we would expect decreased departure likelihood and later nocturnal departure time.

## Methods

### Field work

All birds were caught on Helgoland (54°11′ N, 07°55′ E), a small island ca. 50 km off the German North Sea coastline. Neither wheatears nor song thrushes breed on Helgoland [16], meaning that all birds in this study were actively migrating. Given our intensive (daily) trapping effort, and the strong and often abrupt (daily) changes in the number of migrants, including wheatears and song thrushes, present on Helgoland ([16], CE, TK, HS pers. obs.), we assume that most birds were caught relatively soon after having arrived on Helgoland. We acknowledge that the exact time of arrival at our stopover study site is not known to us, as it is in nearly all stopover studies [50]. However, knowing exact arrival times is not very relevant for the current study, because we assess whether migrants with high oxidative lipid damage are less likely to depart from stopover than conspecifics with little damage. From 25 April to 17 May 2020, wheatears were caught using mealworm-baited spring traps. Traps were monitored continuously, and when a bird was caught it was blood-sampled (ca. 70 μl) from a wing vein within 10 min from triggering the trap. The blood sample was kept on ice until the plasma was separated by centrifugation, which was done within 2 h from capture. The plasma sample was frozen at − 20 °C during the field season and at − 80 °C after that. Following blood-sampling, the birds were ringed, and their subcutaneous fat stores were scored after Kaiser [35] on a scale of 0 (no fat) to 8 (furcular and abdomen bulging, and breast covered with fat). After this, the birds were fitted with a coded radio-transmitter (NTQB2-1 Avian Nano Tag; weight: 0.26 g; Lotek Wireless Inc., Newmarket, ON, Canada; burst interval 2.3–4.7 s) on a leg-loop harness. With the body mass of the wheatears ranging from 20.9–32.3 g, the relative load of the radio-transmitter was always far below the suggested upper permissible load limit of 3–5% of body mass [6, 9]. Radio-transmitters were attached to a Rappole-type harness with the length of the leg-loops adjusted individually to birds [43]. After radio-tagging, the birds were released into the field.

From 19 September to 17 October 2021, song thrushes were caught during routine trapping events performed seven times daily as part of the ongoing ringing of migrants on Helgoland [30]. During these events, birds are caught by chasing them into large funnel traps located in a bushy garden. Birds were blood-sampled (ca. 100 μl) within 10 min from the start of a chase, and the samples were processed and stored as above. The birds were ringed, and fat was scored on the Kaiser [35] scale. Song thrushes were also fitted with a coded radio-transmitter on a leg-loop harness, but the transmitter was a slightly heavier model (NTQB2-1 Avian Nano Tag,weight: 0.41 g; Lotek Wireless Inc., Newmarket, ON, Canada; burst interval 4.7–7.1 s). Because song thrushes are much heavier than wheatears (body mass range of radio-tagged song thrushes: 55.4–80 g), this poses no issue for the suggested upper permissible load limit of 3–5% of body mass. All procedures were approved by the Ministry of Energy, Agriculture, the Environment, Nature and Digitalization, Schleswig–Holstein, Germany (permit number V 244-55709/2018).

### Recording of departure timing

A fully automated telemetry system, as part of the Motus Wildlife Tracking System (see https://motus.org/ and [52]), was used to determine departure timing. This system consisted of four telemetry towers on Helgoland each equipped with a SensorGnome receiver (www.sensorgnome.org) and up to five 6-element Yagi antennas (Vårgårda Radio AB, Vårgårda, Sweden). The 13 antennas in 2020 (similar to [42]) or 16 antennas in 2021 (as in [34]) were radially aligned to provide a full circular coverage. The raw recording data was submitted to Motus through their decoding pipeline, afterwards providing detection data including timestamp, station, antenna direction and signal strength for every individual radio transmitter, i.e. bird. These tracking data were analyzed using an algorithm written by the authors automatically detecting the characteristic departure pattern of a rapid increase in signal strength detected by many antennas, followed by a gradual decline in signal strength from a decreasing number of antennas until the signal is lost, cf. (Fig. 2 in [42]; Fig. 1c,d in [34]). For each radio-tagged bird, we defined departure time as the moment with the highest signal strength during this event (see Fig. 1d in [34]). All data were visually inspected for validation by an independent and unbiased person and if the specific pattern was not clearly present, we did not use the data. This happened for 5 out of 98 radio-tagged wheatears and 12 out of 90 radio-tagged song thrushes.

### Laboratory work

#### Malondialdehyde

Malondialdehyde (MDA) concentration was measured according to Eikenaar et al. [17] by coupled gas chromatography and electron ionization mass spectrometry (GC/EI/MS) analysis after derivatization with O-(2,3,4,5,6-pentafluorbenzyl) hydroxylamine hydrochloride (PFBHA⋅HCl). In brief, 10–15 µl of plasma was mixed with 50 µl pentafluorbenzyl (PFB) solution (1 mM in sodium acetate buffer, pH 5.0) and the micro reaction was conducted for 1 h at room temperature. The resulting MDA-bis-(PFB-oxime) derivatives were extracted by 300 µl n-heptane containing 1.56 pg/µl of 3-bromofluorobenzene as internal standard. The aqueous phase (at the bottom) was removed, and two additional washing steps with 200 µl H_2_O were performed with the aqueous bottom layer being removed between each wash. The final water residual was removed by adding some anhydrous sodium sulfate (Na_2_SO_4_) salt. The remaining extract including the MDA was analyzed by an Agilent 5975C mass-selective detector coupled to an Agilent 7890A gas chromatograph. A non-polar capillary column (HP-5MS: 30 m × 0.25 mm i.d., and 0.25 µm film thickness) was used for GC/MS analysis, and two characteristic ions at *m*/*z* 181 and 250 were measured under selected ion monitoring mode to quantify the target derivatives. The GC oven was programmed from 60 °C for 1 min, at a rate of 15 °C/min to 150 °C, and then at a rate of 10 °C/min to 270 °C, held for 5 min.

#### Total non-enzymatic antioxidant capacity

Total non-enzymatic antioxidant capacity (AOX) was measured (in duplicate) using the ferric reducing antioxidant power (FRAP) assay, which gives the overall reducing potential i.e. non-enzymatic antioxidant potential of the sample [3]. Briefly, 2.5 µl of plasma was diluted 1:8 with ddH_2_O, 20 µl of the diluted plasma sample was then incubated with 150 µl working solution prepared freshly each six hours (Sodium acetate trihydrate + 2, 4, 6-Tris (2-pyridyl)-s-triazine (TPTZ) + Iron (III) chloride hexahydrate (FeCl_3_–6H_2_O); 10:1:1v) for 20 min at room temperature. Immediately following incubation, the colour generated from the reduction of Fe^3+^ (ferric) to Fe^2+^ (ferrous) was measured on a FLUOstar OMEGA (BMG LABTECH) plate reader at 593 nm. The data obtained were compared with a standard curve made from standards of known Fe^2+^ concentration (Iron (II) sulphate heptahydrate (FeSO_4_ – 7H_2_O)) prepared freshly each day.

#### Uric acid

Since the FRAP assay is affected by uric acid (UA) concentration, UA was also measured. UA concentrations were assessed (in duplicate) in 2.5 μl of plasma using a commercial kit from SPINREACT (Sant Esteve de Bas, Spain). Following the manufacturer’s instructions, the red colour, formed after enzymatic (uricase and POD) reactions with uric acid, was measured by a FLUOstar OMEGA (BMG LABTECH) plate reader at 520 nm. In some of the data analyses AOX was corrected for UA concentration (see below). For one wheatear there was not enough plasma to determine UA concentration.

#### Fatty acids

In birds, MDA concentration may to some extent depend on polyunsaturated fatty acid (PUFA) levels (e.g. [45]). For song thrushes, which are much larger than wheatears, we obtained enough plasma to also measure fatty acids (FAs). FAs were extracted and analyzed after base methanolysis using GC/MS according to previously established methods [1]. A total lipid extraction of 5 µl plasma was conducted for 1 h at RT using 50 µl chloroform:methanol (2:1 v/v) containing 33.3 µg of the internal standard methyl *cis*-10-heptadecenoate (purity > 99%, Aldrich). Samples were then dried under N_2_ after which base methanolysis, using 100 µl solution of 0.5 M KOH in methanol, was conducted for 1 h at 40 °C to convert fatty-acyl moieties into corresponding FA methyl esters (FAMEs). The reaction was terminated by adding 100 µl solution of 0.5 M HCl in methanol, and 300 µl *n*-heptane (purity > 99%, VWR Prolabo) was added to extract the resulting FAMEs. Heptane extracts were washed twice with 200 µl de-ionized H_2_O, and residual water removed using anhydrous sodium sulfate. Samples were analyzed using an Agilent 5977B mass-selective detector (MSD) coupled to an Agilent 8890 gas chromatograph (GC) equipped with a polar HP-INNOWax PEG column (30 m × 0.25 mm, and 0.25 µm film thickness; Agilent). Helium was used as carrier gas at a flow of 1 ml/min. The oven temperature was programmed to 80 °C for 1 min, then increased by 10 °C /min to 230 °C and held for 20 min. Nineteen FAMEs were identified by comparing mass spectra and retention times with those of synthetic standards (Supelco 37-Component FAME mix) and quantified based on the abundance of the internal standard.

For every plasma sample, the proportion of each FA was calculated by dividing the peak area by the sum of all FA peak areas. This yielded relative levels of each FA, i.e., the FA composition of a plasma sample. The unsaturation index was calculated as the sum of each FA’s relative level multiplied with their respective number of double bonds [21, 33, 40]. This index reflects the susceptibility of lipids to peroxidation.

All chemicals used were purchased from Sigma-Aldrich (Stockholm, Sweden), unless stated otherwise.

### Data analysis

Statistical analyses were performed using SPSS 27.0 (IBM, New York). Departure decisions were evaluated at two levels. First, we determined departure probability, that is whether or not a bird departed the night after being caught and sampled. We only analysed the first night after capture and sampling, because parameters of the oxidative balance (especially MDA) can change rapidly within days [12, 20, 51]. In other words, for any birds leaving Helgoland later than the day of capture and sampling, we have no information at all about the birds’ oxidative state (or their fat stores). Second, for only those individuals that departed the night after sampling, we determined nocturnal departure time in relation to local sunset (departure in minutes after sunset). Individuals departing on later nights were not considered, as we had no information about their MDA level and AOX on the day of departure.

The first departure level was analysed employing binary logistic regression, with the covariates: MDA concentration, AOX, fat score, and the following weather variables at local sunset on the day of capture: temperature, cloud cover on a scale of 0 (no clouds) to 8 (totally overcast), and the u wind and v wind components, indicative of east–west and north–south wind directions, respectively. Temperature and cloud cover were provided by the local weather station (German Weather Service, DWD). Wind parameters were derived from the database of the National Oceanic and Atmospheric Administration (NOAA); Boulder, CO, USA). Fat score and weather variables were entered as covariates, because these may affect stopover departure decisions [44, 48]. Two models were run, one with raw AOX data, and one in which AOX was ‘corrected’ for UA concentration by entering this as another covariate. The reasons for doing so is that the FRAP assay is affected by UA concentration, and that it is unclear to what extent UA concentration reflects regulated antioxidant defence or merely indicates protein catabolism [10, 17]. For song thrushes (the species for which we measured FAs), the unsaturation index was entered as another covariate. The reason for doing so is that in birds, markers of lipid peroxidation (such as MDA concentration) may to some extent depend on polyunsaturated fatty acid (PUFA) levels [45], but see [21]. The second departure level, nocturnal departure time, was analysed using linear regression with the same set of covariates as used for departure probability. One nocturnal departure time of a wheatear was excluded because the bird departed in the day, ca. 2.5 h before sunset. All statistical analyses were performed separately for the two study species.

## Results

### Departure probability

**Fig. 1:**
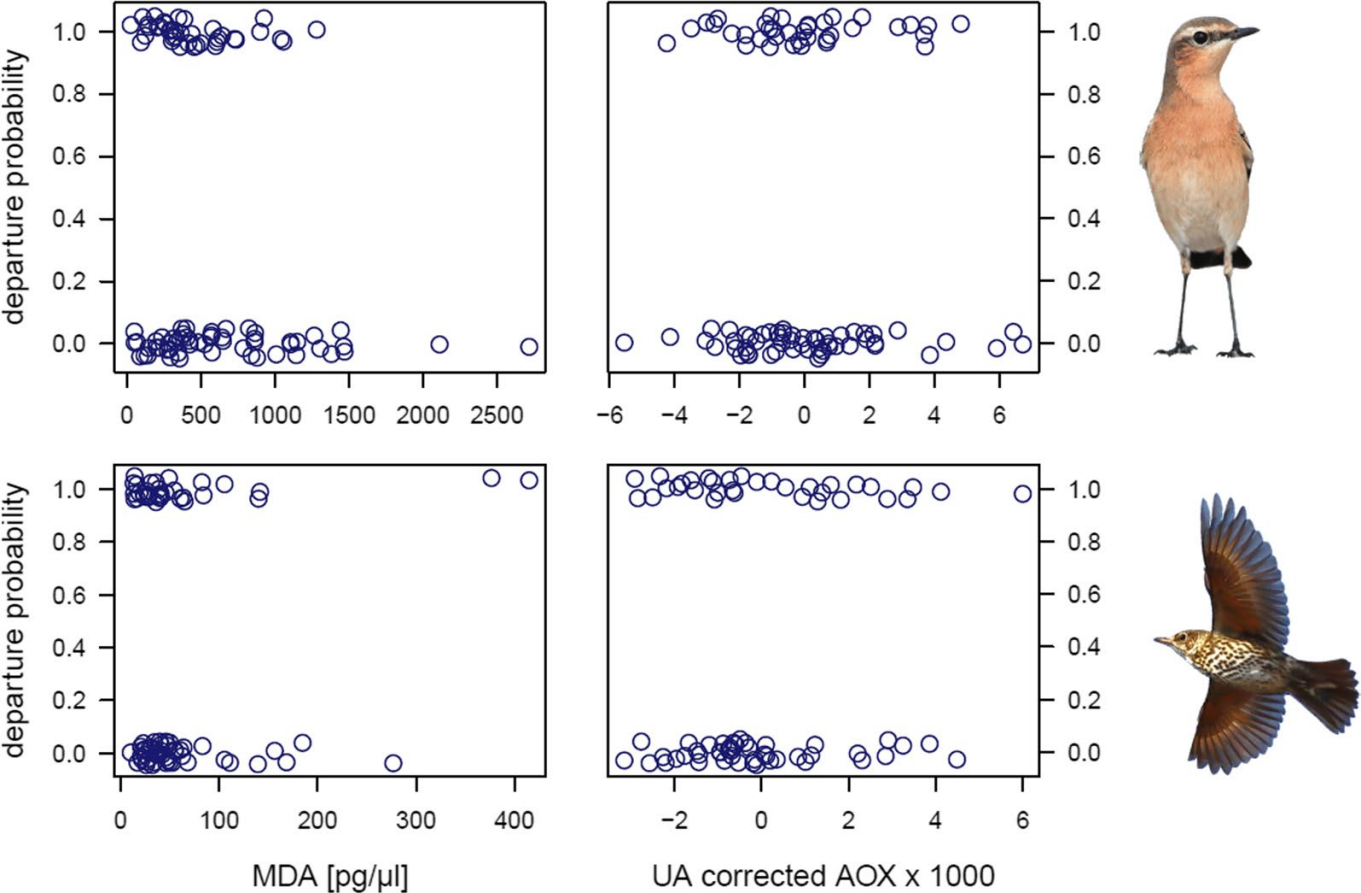
The probability that a migrant departed from our stopover study site the night after capture in relation to its malondialdehyde (MDA) concentration, a marker of oxidative lipid damage, and its total non-enzymatic antioxidant capacity (AOX), corrected for uric acid (UA) concentration. A one means the bird departed and a zero means the bird stayed. The top two panels represent northern wheatears (*Oenanthe oenanthe*) (n = 93 for MDA and n = 92 for UA-corrected AOX) and the bottom two panels represent song thrushes (*Turdus philomelos*) (n = 78). Individual data points are jittered to reduce clumping. To reduce the number of zeros after the decimal point, UA-corrected AOX was multiplied by 1,000. Bird photos by TK

**Fig. 2:**
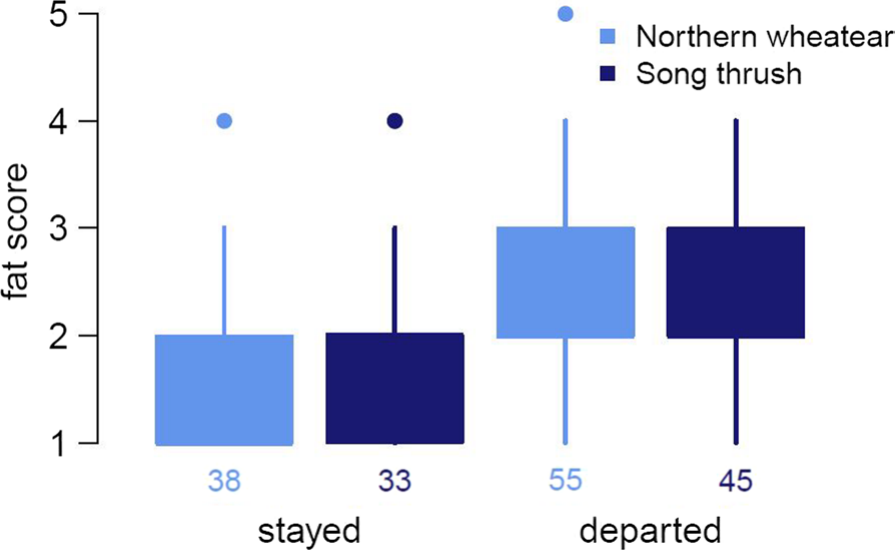
Boxplots of fat scores of staying and departing northern wheatears (*Oenanthe oenanthe*) (light blue) and song thrushes (*Turdus philomelos*) (dark blue). Numbers below boxes represent sample sizes

Of the 93 wheatears, 55 departed in the night after sampling and radio-tagging, and of the 78 song thrushes 45 departed the night after sampling and radio-tagging. Neither in wheatears nor in song thrushes did parameters of the oxidative balance (MDA concentration, FA-corrected MDA, AOX, UA-corrected AOX) affect the decision whether or not to depart in the night after capture and sampling (Table 1, Fig. 1). Weather parameters measured at sunset also did not affect the birds´ departure probability (Table 1). In both species, however, this decision was affected by the birds’ fat stores, with fatter birds being more likely to depart than lean birds (Table 1, Fig. 2).

**Table 1:** The effects of malondialdehyde concentration (MDA), total non-enzymatic antioxidant capacity (AOX), fat stores, and several weather parameters on stopover departure decisions of northern wheatears (*Oenanthe oenanthe*) in spring and song thrushes (*Turdus philomelos*) in autumn on Helgoland

Northern wheatear (n = 93)				Song thrush (n = 78)		
	β ± SE	W	P	β ± SE	W	P
*Without UA*						
MDA	− 0.001 ± 0.001	2.43	0.12	0.002 ± 0.004	0.48	0.49
AOX	− 4.91 ± 65.62	0.01	0.94	41.29 ± 86.81	0.23	0.63
Fat stores	− 0.94 ± 0.31	9.03	0.003	− 0.61 ± 0.29	4.60	0.032
Cloud cover	− 0.15 ± 0.12	1.58	0.21	0.09 ± 0.09	1.03	0.31
Temperature	0.30 ± 0.37	0.67	0.41	− 0.03 ± 0.15	0.04	0.84
u wind	− 0.12 ± 0.14	0.70	0.40	0.01 ± 0.06	0.03	0.87
v wind	0.08 ± 0.08	1.22	0.27	− 0.19 ± 0.14	1.87	0.17
*With UA*				*With UA and UI*		
MDA	− 0.001 ± 0.001	1.76	0.18	0.002 ± 0.004	0.38	0.54
AOX	− 11.02 ± 124.18	0.01	0.93	151.60 ± 156.76	0.94	0.33
Fat stores	− 0.92 ± 0.32	8.19	0.004	− 0.58 ± 0.29	4.04	0.045
UA	2.94 ± 30.17	0.01	0.92	− 38.93 ± 37.31	1.09	0.30
Cloud cover	− 0.16 ± 0.12	1.72	0.19	0.12 ± 0.09	1.51	0.22
Temperature	0.34 ± 0.37	0.86	0.35	− 0.01 ± 0.16	0.01	0.93
u wind	− 0.12 ± 0.14	0.73	0.40	0.01 ± 0.06	0.04	0.84
v wind	0.09 ± 0.08	1.45	0.23	− 0.20 ± 0.14	1.99	0.16
UI				1.04 ± 2.43	1.09	0.30

Binary logistic regression models were run without and with uric acid concentration (UA) as a covariate, and, for song thrush, without and with the unsaturation index (UI) as a covariate. Due to plasma volume limitation, in case of the northern wheatears sample size decreased by one in the latter analysis. Unstandardized coefficients (β) with standard error (SE) and the Wald statistic (W) are presented

### Nocturnal departure time

**Fig. 3:**
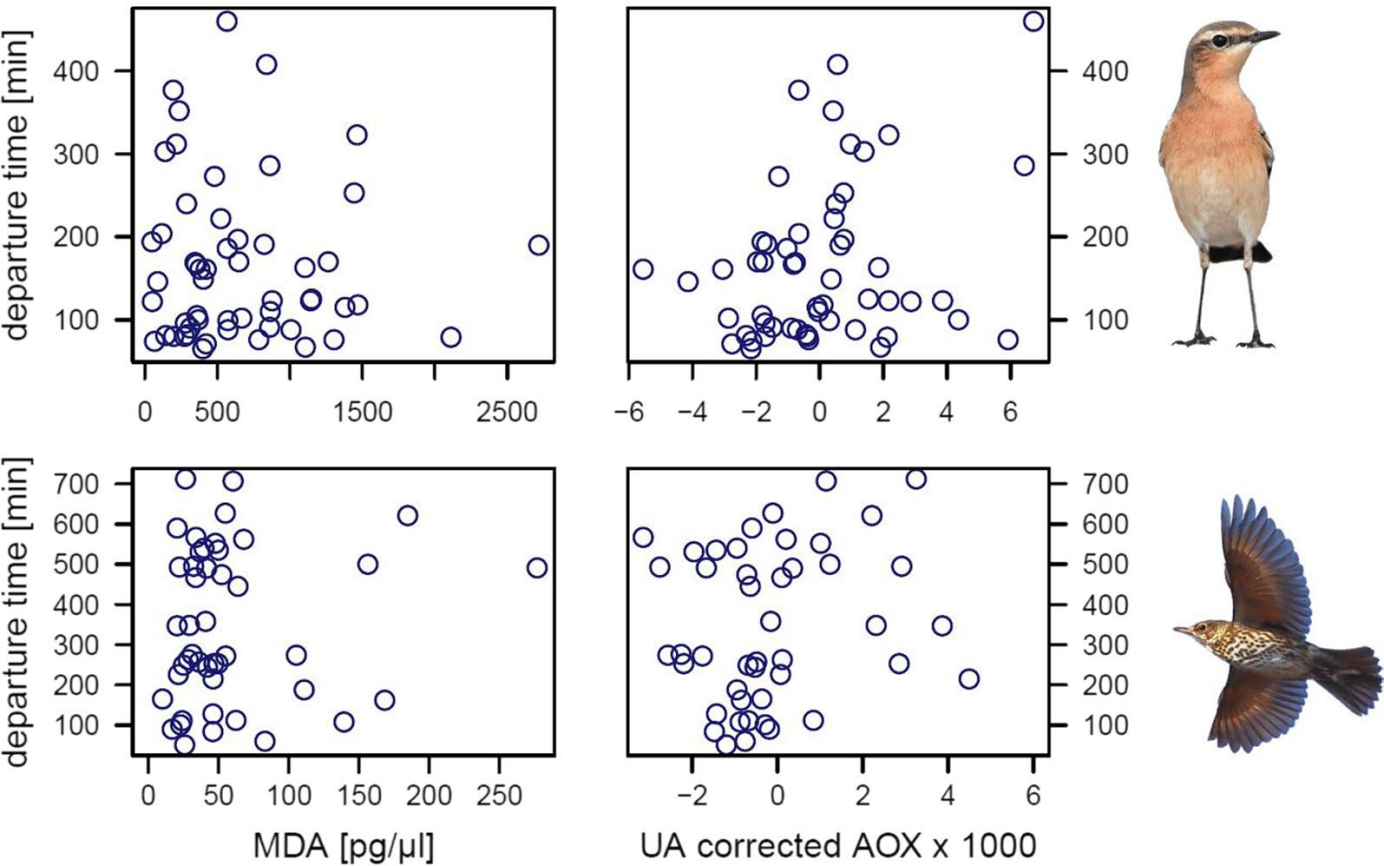
Nocturnal departure time (in minutes after local sunset) as a function of a migrant’s malondialdehyde (MDA) concentration, a marker of oxidative lipid damage, and its total non-enzymatic antioxidant capacity (AOX), corrected for uric acid (UA) concentration. The top two panels represent northern wheatears (*Oenanthe oenanthe*) (n = 54) and the bottom two panels represent song thrushes (*Turdus philomelos*) (n = 45). Note that only individuals which departed the night after capture are plotted in this figure

**Fig. 4:**
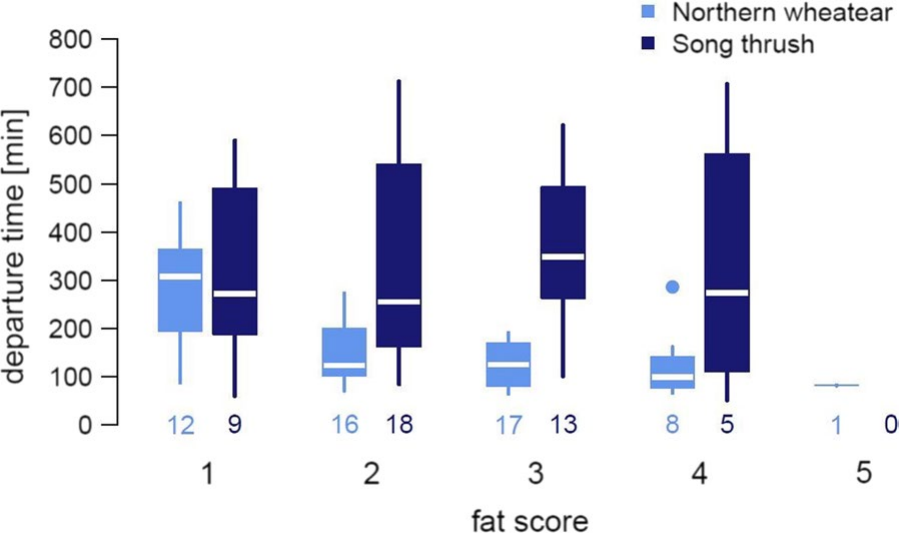
Boxplots of nocturnal departure time (in minutes after local sunset) as a function of fat score in northern wheatears (*Oenanthe oenanthe*) (light blue) and song thrushes (*Turdus philomelos*) (dark blue). Small numbers below boxes represent sample sizes. Large numbers below these represent fat scores

We found no effect of MDA concentration and AOX on nocturnal departure time in either study species, also not when correcting MDA concentration for unsaturation index or when correcting AOX for uric acid concentration (Table 2, Fig. 3). Wheatears departed earlier within the night when carrying larger fat stores, a relationship that was absent in song thrushes (Table 2, Fig. 4). Additionally, wheatears’ nocturnal departure time was negatively related with the v wind component (Table 2), meaning that wheatears departed earlier within the night when experiencing more southerly winds, i.e. wind support. We found no effect in the other weather parameters on wheatears’ nocturnal departure time (Table 2). In song thrushes, weather parameters did not significantly affect nocturnal departure time (Table 2).

**Table 2:** The effects of malondialdehyde concentration (MDA), total non-enzymatic antioxidant capacity (AOX), fat stores, and several weather parameters on nocturnal departure time of northern wheatears (*Oenanthe oenanthe*) in spring and song thrushes (*Turdus philomelos*) in autumn on Helgoland

Northern wheatear (n = 54)				Song thrush (n = 45)		
	β ± SE	t	P	β ± SE	t	P
*Without UA*						
MDA	− 0.01 ± 0.02	− 0.33	0.74	0.20 ± 0.65	0.31	0.76
AOX	3799 ± 2933	1.30	0.20	8449 ± 12,180	0.69	0.49
Fat stores	− 53.10 ± 10.92	− 4.86	< 0.001	17.93 ± 34.40	0.52	0.61
Cloud cover	2.89 ± 4.91	0.59	0.56	12.95 ± 11.09	1.17	0.25
Temperature	18.10 ± 16.10	1.12	0.27	18.43 ± 22.54	0.82	0.42
u wind	− 1.02 ± 6.07	− 0.17	0.87	− 3.30 ± 8.13	− 0.41	0.69
v wind	− 6.78 ± 3.31	− 2.05	0.046	− 22.03 ± 21.06	− 1.05	0.30
*With UA*				*With UA and UI*		
MDA	− 0.02 ± 0.02	− 0.69	0.49	0.24 ± 0.68	0.35	0.73
AOX	8111 ± 4772	1.70	0.096	25,836 ± 25,239	1.02	0.31
Fat stores	− 51.27 ± 11.0	− 4.66	< 0.001	15.70 ± 34.77	0.45	0.65
UA	− 1316 ± 1151	− 1.14	0.26	− 5000 ± 4657	− 1.07	0.29
Cloud cover	3.22 ± 4.90	0.66	0.55	13.54 ± 11.19	1.21	0.23
Temperature	18.46 ± 16.05	1.15	0.26	19.65 ± 23.10	0.85	0.40
u wind	− 2.25 ± 6.15	− 0.37	0.71	− 2.72 ± 8.57	− 0.32	0.75
v wind	− 6.50 ± 3.31	− 1.97	0.056	− 21.32 ± 21.23	− 1.00	0.32
UI				20.43 ± 402.3	− 0.05	0.96

Linear regression models were run without and with uric acid concentration (UA) as a covariate, and, for song thrush, without and with the unsaturation index (UI) as a covariate. Due to plasma volume limitation, in case of the northern wheatears sample size decreased by one in the latter analysis. Unstandardized coefficients (β) with standard error (SE) and the t-statistic (t) are presented

## Discussion

Migratory endurance flight increases oxidative damage [13, 15, 19, 32], which again can be reduced at stopover within a matter of days, at least in case of lipid damage [12, 20, 51]. Restoring the oxidative balance is thought to be one of the many functions of stopover [37, 50]. Although we fully agree with this notion, our current results do not provide much support for this,we found no evidence for an association between migrants’ stopover departure decisions and their malondialdehyde (MDA concentration, indicative of oxidative lipid damage. In a study on temporarily caged migrants, Cooper-Mullin and McWilliams [12] found that supplementation with dietary antioxidants may decrease stopover duration (thus increasing departure probability). However, this effect was only present in one of the two study species, and then only in birds kept on a maintenance diet, i.e. unable to refuel, and not in birds fed ad libitum. Clearly, the role of non-enzymatic antioxidant capacity in migrants’ departure decisions needs further study.

Although finding no effect does not prove the absence of an effect, it is of course possible that migrants’ oxidative state exerts no influence on stopover departure decisions. In that case, the idea that stopovers are made to clear of damages and recover the oxidative balance would be false, and the decrease in oxidative damage observed at stopover [12, 20, 51] is merely a side-effect of decreased metabolic rate, and thus ROS production, from flight to stopover. Although we cannot dismiss this, and in spite of our results, we think this is unlikely because of the high costs of oxidative (lipid) damage, such as repair and replacement of damaged lipids, malfunctioning of cell-membranes [4], and even decreased reproductive output and survival [53]. It seems very plausible that migrants avoid accumulating oxidative damage by making stopovers. We would here also like to again draw the analogy with fuel accumulation (see Introduction); while no-one doubts that this is an important function of stopover, there are quite a few stopover studies which failed to find an effect of fuel stores on departure probability (reviewed in [48]). Perhaps, therefore, more studies are needed to be able to show that stopovers also function to recover the oxidative balance. Touching on this, Schmaljohann et al. [50] recently argued that landing decisions may be more informative when investigating (potential) functions of stopover than departure decisions, the reason being that different individuals likely have a different (set of) reason(s) to make a stopover. In other words, in our dataset there could very well be many individuals that landed and made a stopover for reasons other than reducing lipid damage, for example, they landed to avoid inclement weather. Under such a scenario, studying departure decisions may be inadequate for showing that recovery of the oxidative balance is a function of stopover. Unfortunately, although bio-logging of small migrating birds is rapidly developing [39], studying landing decisions in relation to migrants’ oxidative state is still a bridge too far. Another potential explanation for our negative results may lie in the briefness of stopovers on Helgoland. Each migration season, thousands to tens of thousands of migrants are caught in the Helgoland trapping garden, and many more make a stopover on the island [16]. A potential difference with mainland stopover sites, however, is that the duration of stopovers on Helgoland may be shorter than on the mainland. For example, in the current Helgoland study more than half of the song thrushes departed the night after capture in autumn, whereas at a nearby (German North Sea coastline) mainland stopover site, median autumn stopover duration of song thrushes was 13 days [5]. Perhaps, some migrants leave Helgoland, as part of a landscape movement within the stopover environment [48], and fly to the mainland (ca. 50–100 km) without having (fully) recovered their oxidative balance. Once they have reached their actual stopover on the mainland, they may start (or continue) to restore their oxidative balance. To get an insight in this possibility, it would be necessary to track migrants leaving Helgoland to the mainland and subsequently study the relationship between the oxidative balance and departure decision making there.

In both of our study species, the size of the fuel (fat) stores did have a clear effect on departure decisions. Individuals with larger fuel stores were more likely to depart the night after capture and sampling, and in wheatears, fatter individuals departed earlier within the night than lean individuals. These findings match the general pattern observed in migrants, both on Helgoland (e.g. [44, 46]) and on other stopover sites (e.g. [8, 11, 26, 38]). Perhaps the strong effect that fuel stores have on departure decision making over-rules any (subtler) effect that a migrant’s oxidative state (lipid damage, anti-oxidant capacity) may have on departure behaviour. If so, this could explain why we could not confirm our expectations. Support for this idea comes from the observation that antioxidant supplementation does not affect stopover duration in ad libitum fed and thus fat migrants, while it does in lean migrants kept on a maintenance diet [12]. We are only at the beginning of unravelling the role of physiological parameters, including oxidative balance, on stopover behaviour. Furthermore, not much is known about ‘threshold’ values of physiological markers. For example, how much oxidative lipid damage can a migrating bird handle without this impairing cellular function, behaviour, and ultimately survival? We are confident though, that future in-depth studies will shed new light on the relationships between animal physiology and stopover ecology.

## Conclusions

Stopovers allow migrating birds to accumulate fuel in the form of lipids (fat), and are thought to also allow birds to recover physiologically. One specific type of recovery at stopover is the reduction of oxidative lipid damage, which has been shown to occur in multiple bird species. Perhaps, therefore, recovery of the oxidative state is a function of stopover. In two species of migrating birds, we found that stopover departure decisions were associated with the birds’ lipid stores, but not with the amount of oxidative lipid damage. The latter result provides no further support for the idea that migrating birds make stopovers to improve their oxidative state. That we generally failed to observe an association between stopover departure decision making and migrants’ oxidative state may be explained by an over-ruling effect of the birds’ fuel stores on stopover departure decisions.

## Data Availability

The datasets used and/or analysed for the current study are available from the corresponding author.

## Abbreviations

AOX: Total non-enzymatic antioxidant capacity
EI: Electron ionization
FA: Fatty acid
FRAP: Ferric reducing antioxidant power
GC: Gas chromatography
MDA: Malondialdehyde
MS: Mass spectrometry
ROS: Reactive oxygen species
UA: Uric acid
